# Lipid production and molecular dynamics simulation for regulation of *acc*D gene in cyanobacteria under different N and P regimes

**DOI:** 10.1186/s13068-017-0776-2

**Published:** 2017-04-17

**Authors:** Roshan Kumar, Koushik Biswas, Puneet Kumar Singh, Pankaj Kumar Singh, Sanniyasi Elumalai, Pratyoosh Shukla, Sunil Pabbi

**Affiliations:** 1 Centre for Conservation and Utilisation of Blue Green Algae, Division of Microbiology, ICAR-Indian Agricultural Research Institute, New Delhi, 110012 India; 2 Department of Plant Biology and Biotechnology, Presidency College, Chennai, 600005 India; 3 Department of Biotechnology, Shri JJT University, Jhunjhunu, Rajasthan 333001 India; 4 Enzyme Technology and Protein Bioinformatics Laboratory, Department of Microbiology, Maharshi Dayanand University, Rohtak, Haryana 124001 India; 5 ICAR-National Research Centre on Plant Biotechnology (NRCPB), New Delhi, 110012 India

**Keywords:** Cyanobacteria, Nitrogen, Phosphorus limitation, Lipid production, *acc*D gene expression

## Abstract

**Background:**

Microalgae grown under different nutrient deficient conditions present a good source of natural lipids with applications for several types of biofuels. The expression of acetyl-CoA carboxylase gene can further provide an insight to the mechanisms leading to enhanced lipid production under such stresses. In this study, two nutrients viz. nitrogen and phosphorus were modulated to see its effect on lipid productivity in selected cyanobacteria and its correlation with Accase followed by molecular dynamics simulation.

**Results:**

Selected cyanobacteria viz. *Oscillatoria* sp. (SP8), *Anabaena* sp. (SP12), *Anabaena* sp. (SP13), *Microcoleus* sp. (SP18), and *Nostoc* sp. (SP20) varied in their ability to accumulate lipids which ranged from a lowest of 0.13% in *Anabaena* sp. (SP13) to the maximum of 7.24% in *Microcoleus* sp. (SP18). *Microcoleus* sp. (SP18) also recorded highest lipid accumulation at both N (6 mM NaNO_3_) and P (0.20 mM K_2_HPO_4_) limiting conditions. The overall expression of *acc*D was found to be upregulated in both *Oscillatoria* sp. (SP8) and *Microcoleus* sp. (SP18) for all nitrogen concentrations but was differentially regulated with both positive and negative induction under phosphorus stress conditions. Maximum induction was observed in *Microcoleus* sp. (SP18) at 0.20 mM K_2_HPO_4_. The obtained 3D structure of SP8 protein (21.8 kDa) showed six alpha helices, while SP18 protein (16.7 kDa) exhibited four alpha helices and four beta sheets. The phi (*ϕ*)/psi(*ψ*) angles of the amino acid residues observed in Ramachandran plot analysis showed that both SP8 and SP18 proteins were highly stable with more than 90% amino acids in allowed regions. The molecular dynamics simulation results also indicated the stability of ligand-bound protein complexes.

**Conclusion:**

It has been demonstrated that cyanobacterial isolates are affected differently by nutrient limitation leading to variation in their lipid productivity. The same has been revealed by the behavior of *acc*D gene expression which was regulated more by nutrients concentrations rather than the organism. However, the ligand-bound protein complexes were stable throughout MD simulations.

**Electronic supplementary material:**

The online version of this article (doi:10.1186/s13068-017-0776-2) contains supplementary material, which is available to authorized users.

## Background

Bio-energy production has in recent past become a topic of intense interest due to increased concern over limited petroleum-based fuels supplies and the contribution of the use of these fuels to atmospheric CO_2_ levels. It is further envisaged that with developing world economies, the global energy consumption will also rise leading to more environmental damage. Above all, continued use of petroleum-based fuels and a limited supply has raised a question on its sustainability in near future. Thus, one of the most challenging problems facing mankind today is to find clean and renewable energy sources which are sustainable in the medium to long term. The available alternative sources such as solar, wind, geothermal, wave, and nuclear energy have many benefits but these are generally limited to producing electricity rather than liquid and gaseous energy storage forms and do not provide a solution for other products derived from petroleum. Extensive research has also been carried out to use edible sources like soybean, rapeseed, sunflower, saffola oils [1], and non-edible products as jatropha, greases, tallow, and mahua oils [2–5] as biodiesel; however, higher feed cost is a major bottleneck in its commercial exploitation. Biological systems have the potential to function as feedstock for producing liquid or gaseous fuels and biomass-based fuels are ideal as these are renewable, can achieve high efficiencies and are generally well distributed geographically. Moreover, there are few other related reports for coagulation, flocculation, and sedimentation processes of microalgae which have potential for related studies [6–13]. Among biological systems, many cyanobacteria have the capacity to accumulate substantial amounts of triacylglyceride (TAG) as a storage lipid (ranging from 20 to 50% of cell dry weight) under photo-oxidative stress condition or other environmental stress condition and depending on the species/strain, can also produce different kinds of lipids.

When microalgae are grown in nutrient limited condition, it causes a gradual decline in cell division rate; however, the fatty acids biosynthesis is maintained under such conditions [14]. When the cell growth is slow, the synthesis of new compounds stops but the microalgae accumulate fatty acids and TAG and this works as a protective mechanism. In normal growth condition, ATP and NADPH produced during photosynthesis are used for biomass production and ADP and NADP^+^ are finally made available again for photosynthesis as acceptor molecules. In case, when cell growth and multiplication is impaired due to nutrient deprivation, the pool of NADP^+^ decrease leading to formation of NADPH which is used for fatty acid biosynthesis (which in turn are stored in TAGs) thus replenishing the pool of NADP^+^ [14–16]. Nitrogen manipulation plays a critical role for high lipids production [17–22] and is also cost effective compared to other factors. Some microalgae like *Chlorella* sp., *Nannochloropsis* sp., *Neochloris oleoabundans*, *Botryococcus braunii* accumulate highest amount of lipids under nitrogen starvation [23] and considered to be one of the best conditions for the enhancement of lipids, SFA and MUFA in microalgae [24]. Nevertheless, nitrogen stress might not always promote lipid induction but can result in change in lipid composition as well with accumulation of fatty acids like C16:0, C18:1, C18:2, and C18:3 [25, 26]. Similarly, phosphate limitations have been shown to induce lipid accumulation mainly as TAGs in *Phaeodactylum tricornutum, Chaetoceros* sp*., Isochrysis galbana,* and *Pavlova lutheri,* but decrease in *Tetraselmis* sp. [15, 27]. In phosphate-deprived medium, C16:0 and C18:1 fatty acid production was enhanced whereas other fatty acids like C18:4ω3, C20:5ω3, and C22:6ω3 decreased [27].

## Fatty acid biosynthesis and acetyl-CoA

The first important committing step in lipid biosynthesis begins with acetyl-CoA carboxylase (ACC). It catalyzes the biotin-dependant carboxylation of acetyl-CoA to form malonyl-CoA. Carbon of all fatty acids is made available from the pool of acetyl-Coenzyme A (CoA) present in the plastid and act as precursor for fatty acid synthesis pathway. The acetyl-Coenzyme A concentration in chloroplast is only 30–50 µM [28] which is enough for the biosynthesis of fatty acids. However, the acetyl-CoA pool should remain relatively constant, even when fatty acid synthesis rates vary greatly, depending on light (when synthesis is relatively high) and dark (when synthesis is low). Thus, a system must be there in plastids that quickly produces acetyl-CoA for continuous fatty acid production. There are multiple pathways which are involved in higher lipids production in microalgae and transcriptomics serves as an excellent tool to provide initial, broad idea of carbon partitioning, and regulation of TAG biosynthesis in microalgae during stress responses [29]. However, the most potential strains identified so far by growth experiments and lipid content have not been sequenced and do not have fully annotated genomes [30–34]. Today transcriptomic studies are mainly focused on specific microalgae which have fully sequenced genome compared to oleaginous [35, 36] and a number of genes in central carbon metabolism and TAG biosynthesis pathway have been monitored to know, how cell are adapting in stress environment with changes in molecular level that induce TAG hyperaccumulation [37, 38]. Cronan and Waldrop [37] reported that nitrogen, phosphorous, and iron limitation also trigger the increase of *acc*A and *acc*D gene expression level in microalgae [39]. However, studying the lipid content and expression analysis of three genes (involved in carbon fixation and lipids biosynthesis pathway) viz. homomeric/eukaryotic acetyl-CoA carboxylase (*acc*1 gene), heteromeric/bacterial acetyl-CoA carboxylase (ACCase) beta subunit (*acc*D gene), and ribulose 1,5-bisphosphate carboxylase/oxygenase (RuBisCO) large subunit (rbcL gene) in *Chlorella Sorokiniana* CCTCC M209220 strain found that the expression level of all three genes were increased in stationary phase than logarithmic phase at different glucose concentration (10 g/l).

In the recent years, the prediction of insilico three-dimensional protein structures of various enzymes has enabled us to find the catalytic potential of particular enzyme. These three-dimensional modeled structures of protein imitate the interaction of enzyme substrate to provide the catalytic efficiency of enzymes [40, 41]. This progress has occurred due to new methodology to gather large amount of information in sequence and structure database and improved computational descriptions of protein energetics [42]. Metabolic network reconstruction provides avenue to design different metabolic reactions to maximize the growth of particular byproduct. Apart from this, metabolic reconstruction also helps to reveal the inhibiting factors [43–48]. Furthermore, there are recent reports on utilizing gene editing, novel platform technologies which are quite evident towards developing novel insights about gene expression levels [49, 50].

## Methods

### Microorganism and experimental design

Five cyanobacterial strains viz. *Oscillatoria* sp. (SP8), *Anabaena* sp. (SP12), *Anabaena* sp. (SP13), *Microcoleus* sp. (SP18), and *Nostoc* sp. (SP20) isolated from the soil of different regions of India viz. Tamil Nadu, Haryana, Delhi, Kashmir, and Bihar and conserved in germplasm center at Centre for Conservation and Utilisation of Blue Green Algae, IARI, New Delhi, India were used for the study. The strains were maintained in BG-11 medium [51] in a culture room under cool white fluorescent light intensity of 50–55 μmol photons/m^2^/s with photoperiod of 16:8 h light/dark at 28 ± 2 °C. The 14-day-old culture was used as inoculum and a 2% inoculum was used to inoculate the experimental flasks. The cultures were studied for production of total lipids at different incubation periods viz. 7, 14, 21, 28, and 35 days. For stress-related studies, the observations were made after 28 and 35 days of incubation when lipid production was maximum. The cultures were grown in different sets with two different sets each for five NaNO_3_ concentrations (3, 6, 9, 12, and 15 mM) and two different sets each for five K_2_HPO_4_ concentrations (0.05, 0.10, 0.15, 0.20, and 0.25 mM) for nitrogen and phosphorous stress, respectively, by supplementing in normal media and under normal conditions of temperature and light. One set of cultures was removed at the end of each incubation period (28th and 35th days after inoculation) and analyzed for biomass [52] and total lipids [53]. The lipid samples were processed for preparation of fatty acid methyl esters (FAME) [54] and fatty acid profile for each of the experimental conditions was determined using gas chromatography (Shimadzu GC-2010) as described by [55].

### PCR based molecular detection of acetyl-CoA carboxylase

The genomic DNA isolated from the cyanobacterial cultures was used as template to amplify Acc carboxylase gene which is well known for active involvement in fatty acid synthesis pathway and also in biofuel production using gene specific primers with the help of PCR. Two sets of gene specific degenerate primers were designed (SP8-PCR and SP18-PCR) (Additional file 1: Table S1) based on the available gene sequence information in National Center for Biotechnology Information database (NCBI) using Integrated DNA Technology (IDT) online tool. The PCR reaction was performed in a total volume of 25 µl having designed primers (10 mM, 0.5 μl each), dNTPs (25 mM, 0.25 μl), MgCl_2_ (1.25 μl), DNA polymerase buffer (10x, 2.5 μl), and 1U of Taq Polymerase (Merck, USA). Amplification was achieved in an automated thermal-cycler (GeneAmp PCR9700 system, Applied Biosystems, USA) set for initial denaturation (94 °C for 3 min.) followed by 35 cycles comprising of denaturation (95 °C for 1 min.), annealing (56 °C for 1 min.) and extension (72 °C for 45 s) followed by a final extension of 5 min at 72 °C. PCR product was subjected to further purification using QIAquick PCR purification kit protocol (Qiagen, USA) for nucleotide sequencing. Amplicons of sizes 924 and 1142 bp obtained for strains SP8 (*Oscillatoria* sp.) and SP18 (*Microcoleus* sp.), respectively, (Fig. 3) were sequenced. The obtained nucleotide sequences were analyzed by NCBI-BLAST sequence similarity search and submitted to EMBL-EBI database (Accession No. LN606590 and LN606589) (Additional file 1: Table S2).

### RNA isolation and q RT-PCR based expression profiling of acetyl-CoA carboxylase

Two selected strains of BGA viz. SP8 (*Oscillatoria* sp.) and SP18 (*Microcoleus* sp.) were taken, and 35-day-old culture grown under five different concentrations of K_2_HPO_4_ and NaNO_3_ was used for isolation of total RNA using Trizol RNA extraction procedure (Invitrogen, USA). Total RNA was quantified with Nanodrop spectrophotometer (Thermo Scientific NanoDrop 2000C Technologies, Wilmington, USA) and the integrity of total RNA was checked by 1.2% agarose gel electrophoresis and ethidium bromide staining. 1 μg of each RNA sample was used for first-strand cDNA synthesis using SuperScript™ III First-Strand Synthesis System protocol (Invitrogen, USA). The qPCR reaction was performed with the synthesized cDNA as template. Based on the sequence information of homologs ascertained from SP8 and SP18 isolates (Accession No. LN606590 and LN606589), two different sets of qPCR primers for Acc carboxylase gene were designed (Additional file 1: Table S2) for expression profiling at variable stress conditions. The reaction mixture contained 2x KAPA SYBR^®^ FAST qPCR Master Mix, (2x–10 μl) (Wilmington, USA); Pimers (10 mM–1 μl each); cDNA template, (40 ng/μl–5 μl); and PCR grade water (3 μl) and reaction was performed in LightCycler^®^ 480 Real-time RT-PCR system (Roche Diagnostics GmbH, Penzberg, Germany) set for 40 cycles with initial denaturation at 95 °C for 3 min, denaturation at 95 °C for 10 s, annealing temperature at 55 °C for 30 s, extension temperature at 72 °C for 1 s, with an additional melting temperature having ramp from 65 to 95 °C. For endogenous control, constitutively expressed 16S-rRNA gene was used as an internal standard to normalize differences between the loading intensity of target and reference gene. The experiments were repeated twice independently, and the fold change data were taken as average. All events were performed according to the manufacturers’ directions. The 2−ΔΔCT method was used to analyze the fold change in gene expression [56].

### Insilico analysis

Gene predictions in SP8 and SP18 sequences were performed by FGENESB (http://linux1.softberry.com/) after editing the sequences through BioEdit v.7.2.3 software [57]. The active site prediction was performed by ScanProsite tool of ExPasy (http://prosite.expasy.org/cgi-bin/prosite/). Predicted proteins of both sequences SP8 and SP18 were used for 3D modeling and structure prediction. The 3D models were predicted by both ways based on homology and ab initio modeling. The homology-based models were built by SWISS-MODEL (http://swissmodel.expasy.org/) using default parameters. The 3D models of SP8 and SP18 were generated by ab initio structure prediction method using I-TASSER web-based server (http://zhanglab.ccmb.med.umich.edu/I-TASSER/). The 3D structures and predicted models were visualized using RasMol v.2.7.5.2 (http://www.rasmol.org/) and UCSF Chimera v.1.9 [58] softwares. The later software was also used in the analysis of 3D conformational structural comparison between SP8 and SP18 proteins and generation of Ramachandran plot of the proteins. The models were also tested by PDBsum server (http://www.ebi.ac.uk/thornton-srv/databases/pdbsum) to get detailed structural information of SP8 and SP18 proteins. The physicochemical properties of the protein sequences of both genes SP8 and SP18 were calculated using Protparam software (http://web.expasy.org/protparam/). The QMEAN server (http://swissmodel.expasy.org/qmean/cgi/index.cgi/) was used to find the overall quality of three-dimensional structure of SP8 and SP18 proteins. The predicted 3D models were subjected for docking with Biotin protein (1C2Q.pdb) retrieved from protein data bank (http://www.rcsb.org/pdb/home/home.do/). The unbound docking was performed by ZDOCK 3.0server (http://zdock.umassmed.edu/) to check their all possible interactions.

### Statistical analyses

The data (in triplicate sets) for the parameters evaluated were subjected to ANOVA (analysis of variance) according to the experimental design (completely randomized design) using the statistical package MSTAT-C to quantify and evaluate the source of variation, and CD (critical differences) values (calculated at *P* level of 0.05). Alphabetical superscripts in tables denote ranking in descending order, i.e., a denotes the highest value according to Duncan’s multiple range test (DMRT) and similarity matrix. For studying the similarity/dissimilarity between the cyanobacterial strains, cluster analysis was performed using SAS 9.3. The Dendrogram was constructed using unweighted pair-group method using arithmetic averages (UPGMA) based on Euclidean distance. Three basic statements were used. viz. PROC DISTANCE (to calculate the distance matrix between species), PROC CLUSTER (to calculate the clusters) and PROC TREE (to draw the dendrogram).

### Molecular dynamic simulation

The simulation was executed with GROMACS v. 4.5 package with the force field as Gromos43a1. The PRODRG server was used to prepare the necessary coordinate and topological files with GROMACS compatibility. The ligand and protein salvation was executed with SPC water model in a cubic box (10.8 × 10.8 × 10.8 nm^3^). Here, the Counter-ion (1 Cl^−^) was included to counterbalance the solvated system. With the aim to guarantee that there are negligible steric clashes or improper geometry in such solvated system of protein–ligand complex, it was processed for energy minimization using the steepest algorithm up to a maximum 25,000 steps or until the maximum force (Fmax) is not greater than 1000 kJ/mol nm which is the default threshold. The quantity of steps necessitated to reach minimization criteria were scrutinized to be around 400 for such complex. This step was performed after the equilibration of the complete system by means of both NVT/ and NPT ensembles for 50,000 steps (100 ps) at 300 K and 1 atm. Here, the system was firstly equilibrated using NVT ensemble followed by NPT ensemble. After this equilibration process, the molecular dynamics simulation was carried out for 2 ns long. Finally, the simulations were evaluated, and RMSD, RMSF, and hydrogen bonds plots were calculated for complete episode of simulations.

## Results and discussion

### Total lipids

Total lipids from the cyanobacterial strains viz. *Oscillatoria* sp. (SP8), *Anabaena* sp. (SP12), *Anabaena* sp. (SP13), *Microcoleus* sp. (SP18), and *Nostoc* sp. (SP20) were measured gravimetrically and expressed as dry weight percentage (Table 1). Maximum amount of lipids was accumulated at 35 days which was a gradual increase from day 7 till the end of incubation. Total lipids production in all the isolates varied and ranged from a lowest of 0.14% in *Anabaena* sp. (SP13) to the maximum of 7.24% in *Microcoleus* sp. (SP18).

**Table 1:** Time course study of total lipid production in selected cyanobacteria

S. no.	Cyanobacteria	Total lipid production (% dry weight) after days of incubation				
		7	14	21	28	35
1.	*Oscillatoria* sp. (SP8)	0.36	1.21	2.17	2.64	4.85
2.	*Anabaena* sp. (SP12)	0.38	1.58	3.17	4.63	5.31
3.	*Anabaena* sp. (SP13)	0.14	2.54	4.00	4.75	5.34
4.	*Microcoleus* sp. (SP18)	1.42	3.48	4.15	5.19	7.24
5.	*Nostoc* sp. (SP20)	1.52	2.58	3.74	5.30	6.57
	CD@5%	*0.554*	*1.552*	*0.382*	*0.241*	*0.321*

**Fig. 1:**
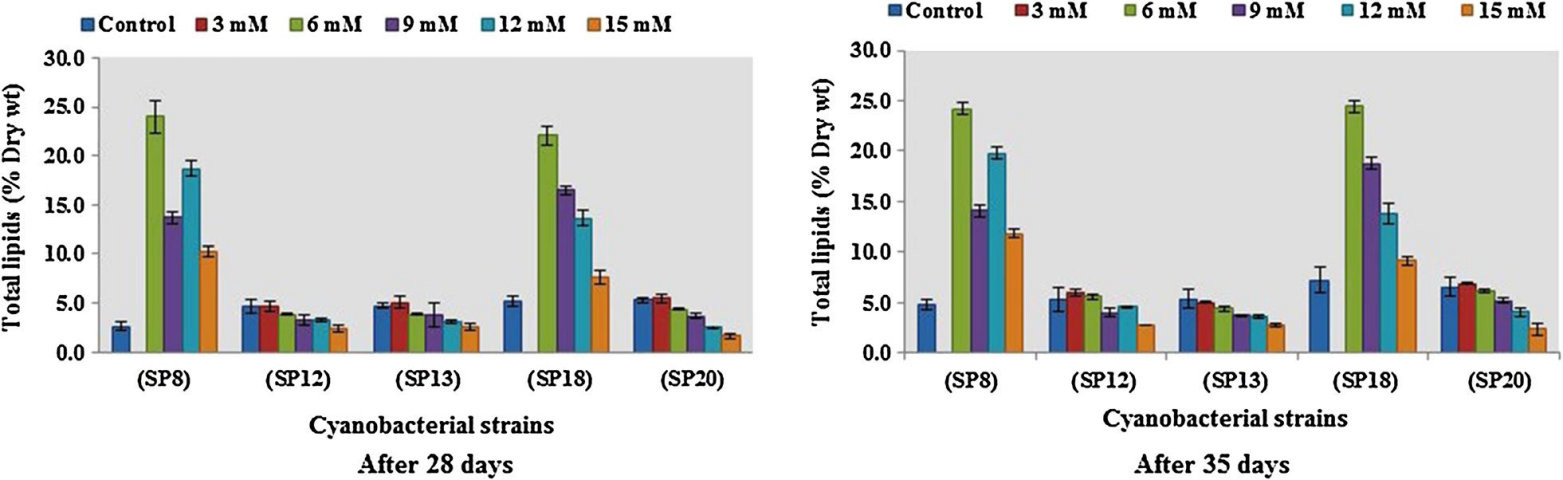
Total lipid production in selected cyanobacteria grown under different nitrogen (NaNO_3_) regimes after 28 and 35 days of incubation

**Fig. 2:**
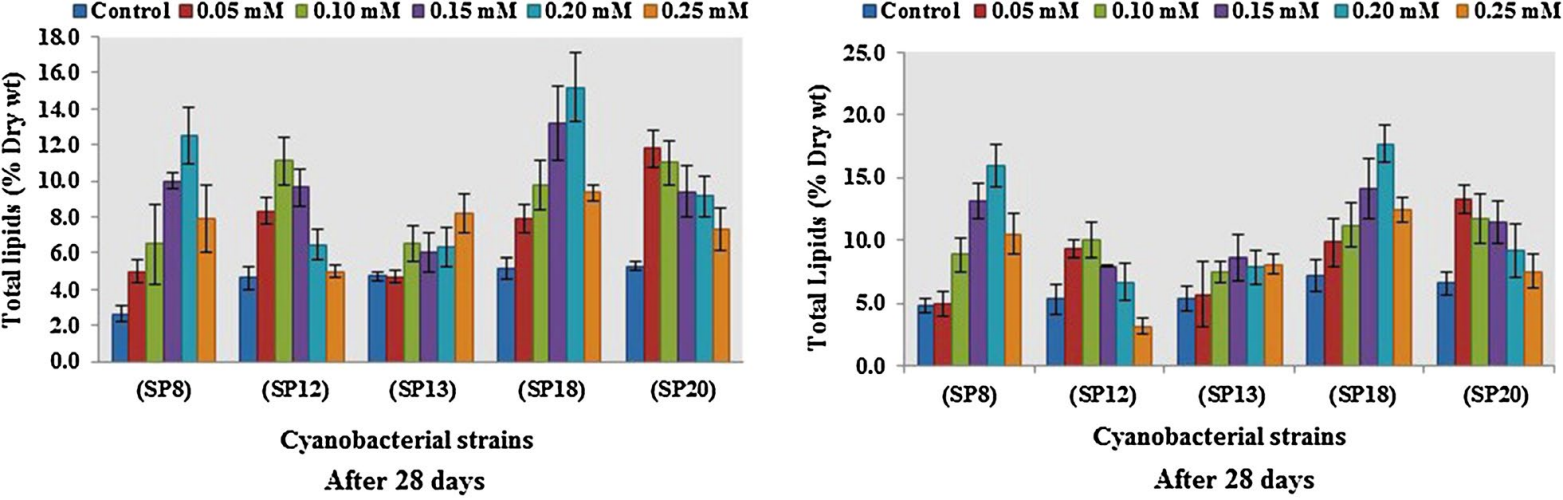
Total lipid production in selected cyanobacteria under different phosphorous (K_2_HPO_4_) regimes after 28 and 35 days of incubation

To investigate the effect of varied nitrogen and phosphorus concentrations on total lipids production, the biomass and lipid accumulation was evaluated after 28 and 35 days duration (Figs. 1, 2). It was very interesting to note that *Oscillatoria* sp. (SP8) and *Microcoleus* sp. (SP18) which did not survive at lower nitrogen concentration of 3 mM showed maximum fold increase in total lipid accumulation when grown at 6 mM nitrogen. All other cultures showed variable level of total lipids at different N concentration. Total lipids showed the increasing trend from 10.2 to 23.9%, 2.46 to 4.62%, 2.52 to 5.10%, 7.67 to 22.2%, and 1.63 to 5.57% at 28 days of incubation and 11.8 to 24.2%, 2.73 to 5.98%, 2.83 to 5.03%, 9.14 to 26.8%, and 2.37 to 6.89% at 35 days in *Oscillatoria* sp. (SP8), *Anabaena* sp. (SP12), *Anabaena* sp. (SP13), *Microcoleus* sp. (SP18), and *Nostoc* sp. (SP20), respectively. *Microcoleus* sp. (SP18) and *Oscillatoria* sp. (SP8) recorded highest lipid accumulation under 6 mM nitrogen. Maximum percentage increase of 26.8% in lipid production was achieved in *Microcoleus* sp. (SP18) at 6 mM nitrogen concentration after 35 days of incubation (Fig. 1). Lipid accumulation in cyanobacterial strains at different levels of phosphorus viz. 0.05, 0.10, 0.15, 0.20, and 0.25 mM increased by varied amounts as compared to control i.e., from 4.49 to 12.5%, 4.99 to 11.1%, 4.70 to 8.19%, 7.94 to 15.2%, and 7.32 to 11.8% at 28 days of incubation and 5.67 to 15.9%, 3.17 to 10.0%, 5.71 to 8.85%, 9.73 to 17.7%, and 7.53 to 13.2% at 35 days of incubation in *Oscillatoria* sp. (SP8), *Anabaena* sp. (SP12), *Anabaena* sp. (SP13), *Microcoleus* sp. (SP18), and *Nostoc* sp. (SP20), respectively. Maximum lipid productivity increase of 153% was again shown by *Microcoleus* sp. (SP18) at 0.20 mM phosphate concentration after 35 days of incubation (Fig. 2). Consistent with a number of studies, the modulation of N and P nutrition resulted in increase in total lipid accumulation [31, 38, 59]. However, the current literature provides fragmented information regarding the strategies for lipid production improvement, the one that is most commonly applied is the design of nutrients viz. nitrogen, phosphorus, and sulfur starvation or limitation. Ito et al. [60] reported that under nitrogen stress conditions, the quantities of neutral lipids in micro algal cells were greatly increased, while amino acids were significantly reduced to 1/20 of the amount or even less. Mandal et al. [61] cultivated the microalgae *Scenedesmus obliquus* under phosphorus starvation and witnessed a lipid content increase from 10.0 to 29.5%. Temperature modulation along with nutrient stress is another strategy which not only enhances lipid productivity but also affects the composition of lipids produced. With nitrogen deprivation, the total and neutral lipids increased with increasing temperature, and the highest lipid content of 47.60% of cell dry weight and the highest TAG content of 79.66% of total lipid was achieved at 33 °C. Thus, high temperature induced carbon flux from starch towards TAG accumulation in microalgae during nitrogen starvation and further reduced the degree of the fatty acid unsaturation and favored a better biodiesel production [62].

It is of particular significance in large scale microalgal lipid production processes suggesting that nitrogen and phosphorus starvation strategy is most favorable for lipid productivity.

### Expression study

For molecular detection of Acc carboxylase gene, PCR amplification was performed using two distinct sets of designed degenerate primers (SP8-PCR and SP18-PCR) (Additional file 1: Table S1) and amplicons of sizes 924 and 1142 bp obtained for strains SP8 (*Oscillatoria* sp.) and SP18 (*Microcoleus* sp.), respectively, (Additional file 1: Figure S1) were sequenced. The obtained nucleotide sequences were analyzed by NCBI-BLAST sequence similarity search and submitted to EMBL-EBI database (Accession No. LN606590 and LN606589) (Additional file 1: Table S2). The sequence obtained from SP8 showed similarity with *Oscillatoria acuminate* PCC 6304 chromosome (complete genome) with percent identity of 72% and query coverage up to 56%, whereas the sequence generated from SP18 displayed maximum similarity with *Microcoleus* sp. PCC 7113 chromosome (complete genome) with identity percentage of 73 and query coverage up to 47%. In both cases, the results were found to be exactly similar with the cyanobacterial strains which were initially taken for molecular studies.

**Fig. 3:**
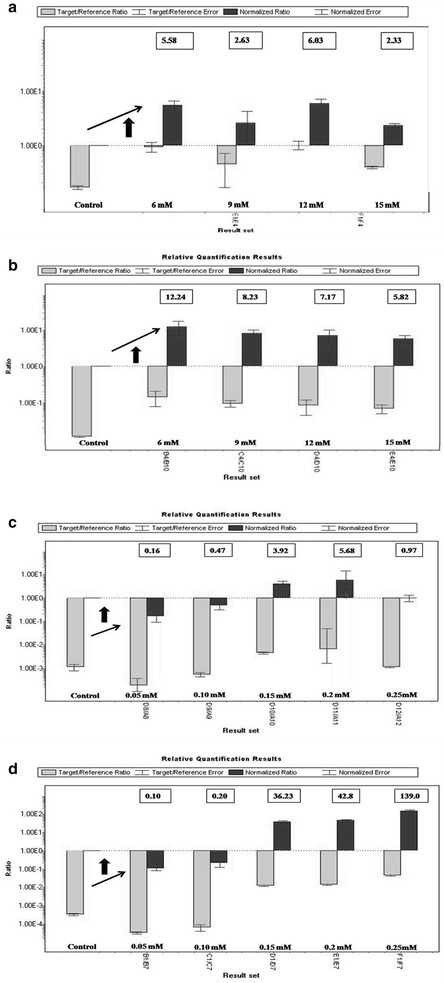
Real-time expression profile of Acetyl-CoA carboxylase gene under nutritional stress (nitrogen and phosphorus). **a** *Oscillatoria* sp. (SP8) under NaNO_3_ stress, **b** *Microcoleus* sp. (SP18) under NaNO_3_ stress, **c** *Oscillatoria* sp. (SP8) under K_2_HPO_4_ stress, **d** *Microcoleus* sp. (SP18) under K_2_HPO_4_ stress

The expression levels of acetyl-CoA carboxylase gene in *Oscillatoria* sp. and *Microcoleus* sp. were studied under five limited doses of nitrogen and phosphorus using real-time PCR during stationary phase of their growth (Fig. 3). The overall expression of the Acc carboxylase gene related to lipid synthesis in *Oscillatoria* sp. and *Microcoleus* sp. was found to be upregulated for all limited nitrogen stress conditions. In contrast, expression levels of this gene under limited phosphorus stress conditions were differentially regulated with a combination of positive and negative induction. However, the level of gene expression could not be determined under very limiting nitrogen stress condition with a lowest concentration of 3 mM since at that level the algal cultures failed to survive. This is due to the fact that nitrogen is a basic nutritional source for BGA growth and multiplication and is required with a minimum threshold limit.

The effect of nitrogen limited condition on gene expression in *Oscillatoria* sp. is quite interesting which has shown a variable range of positive induction with a gradual increase of NaNO_3_ supply. Initially after immediate increase of NaNO_3_ concentration from 3 to 6 mM, the Acc carboxylase gene is expressed up to 5.58-fold compared to the control which confirmed that the said enzyme get involved in stimulating the initiation of lipid synthesis at ground level. As the nitrogen concentration increased from 6 to 9 mM, the relative expression of the gene was down with apparent fold change (FC) of 2.63 (Fig. 3). It is quite possible that after immediate activation of this enzyme, carboxylase gene product might have accumulated in sufficient amounts to necessitate the switching off of the gene and a continuous increase of nitrogen in the metabolic system further accelerated gene activity gaining induction up to 6.03-fold from near to ground level (FC-2.63) under 12 mM concentration of NaNO_3_. The expression of Acc carboxylase finally downregulated to 2.33-fold at the terminal point of highest nitrogen abundant condition (Fig. 3). The overall expression pattern of the gene in *Oscillatoria* sp. showed zigzag type model and it could be possibly explained by the reasoning that most gene expression is dynamic in nature which is not expected to be upregulated in all points of factor. Wan et al. [39] also reported a similar type of expression behavior of this gene under nitrogen stress condition in *Chlorella sorokiniana*. On the other hand, the gene expression pattern in *Microcoleus* sp. under nitrogen stress condition was considerably different than in *Oscillatoria* sp. showing a gradual step down of induction as the NaNO_3_ concentration changed from lower to upper limit. At initial supply of NaNO_3_ (6 mM), the gene instantly got upregulated and its expression increased up to 12.24 in terms of FC (Fig. 3) which represents the highest level of stimulation of the gene product under nitrogen stress condition as per conducted study, thereby confirming that the enzyme is solely involved and catalyzed maximum fatty acid biosynthesis reaction under lower limit of nitrogen supply. As the nutrient concentration is proportionately increased from 6 mM up to 15 mM, the expression level continuously decreased to a final value of 5.82-fold (Fig. 3). Overall, the enzyme performed better under limited nitrogen condition in *Microcoleus* sp. than in *Oscillatoria* sp., Huerlimann et al. [63] while studying the effect of nitrogen limitation on Accase expression reported its significant upregulation during nitrogen starvation and culture age in *Chromera velia,* whereas it was not differentially expressed in *Isochrysis* aff. *galbana* (TISO) but got upregulated during the logarithmic phase of nitrogen replete cultures. The study concluded that Accase changed with nutrient status and culture age in a species specific manner and nitrogen limitation did not always result in fatty acids accumulation [63]. The overall expression pattern of Acetyl-CoA carboxylase (ACCase) actively involved in the fatty acid biosynthetic pathway might be controlled by the individual regulation behavior of its three subunits viz. *accA*, *accD*, and *bccp* [64]. Therefore, it is quite possible that the *acc* gene expression outline is not only inclining under nitrogen stress gradient but sometimes do not follow a specific up- or downregulation pattern under various salinity stress conditions in oleaginous culture [65].

The effect of phosphorus concentration on Acc carboxylase gene expression is quite different from nitrogen and it was a combination of positive and negative regulation of gene function under variable ranges of stress points of K_2_HPO_4_ supply. When the phosphorus stress was imposed with limited quantity in *Oscillatoria* sp., the gene is slightly downregulated with a fold change of 0.16 at initial concentration of K_2_HPO_4_ (0.05 mM). As the phosphorus availability is gradually increased, the induction of the gene is turning from negative to positive with a steady enhancement viz. 0.47, 3.92, and 5.68 (Fig. 3). However, at highest concentration of K_2_HPO_4_ (0.25 mM), the expression suddenly dropped down reaching almost the point of base level (FC-0.97). Phosphorus is considered to be a vital constituent of ATP for photophosphorylation which is required preferably more than a certain limit to stimulate the gene activity involved in metabolic pathway such as lipid biosynthesis. Therefore, there is a chance that the associated genes are negatively induced below a threshold point of phosphorus supply and this point could be lying between 0.10 and 0.15 mM for Acc carboxylase as per results of this study (Fig. 3). The expression profile of the gene under study in *Microcoleus* sp. in phosphorus deprivation condition presented similar results as observed in *Oscillatoria* sp. However, in addition, the induction pattern gradually increased from negative to positive point ending at 139.0 (0.25 mM) fold which can be considered as the highest level gene expression under overall study (Fig. 3). In one of the studies [66], the total lipids in *Chlorella* sp. was up to 23.0% of cell dry wt. (CDW) under normal P concentration and after exposure to 25% reduction, it declined to 18.0% of CDW, while 50% reduction resulted in insignificant changes (*P* > 0.05) in total lipids as compared to control. A further reduction by 75% resulted in a significant increase of total lipids up to 32% of CDW thus showing a variable response at different reductions. A comprehensive study on the effects of phosphorus limitation versus starvation on gene expression of *Prochlorococcus* MED4 II revealed that while there were changes in expression of P-uptake genes, the sulfolipid synthesis gene (*sqdB*) expression was significantly lower under P-limited growth than either P-replete or P-starved condition while there was no change in the expression of phospholipid synthesis gene (*pgsA*) under P-replete, limited or starved condition [67]. Overall, in the present study, expression levels of the gene has given us an idea that effect of nitrogen has little correlation with lipid content because expression levels of Acc carboxylase gene were consistently low in both the cultures but phosphorus has dramatic influence with higher degree of induction. On the other hand, it can also be concluded that the stationary phase of both algal species is directly associated with the high expression of the said gene and that is equally important for accumulation and synthesis of lipids. Several studies on relative quantification of Acc carboxylase gene affirmed that stationary phase is more promising than log (exponential) phase in terms of higher gene expression. The relative expression of *acc*D gene in *Chlorella sorokiniana* under nitrogen deprivation conditions suggested that the gene induction level was low in log phase in all the cultures [39]. Similar results were also found in another study where effects of mixotrophy were correlated with microalgal growth, lipid production, as well as expression of three pathway genes including *acc*D gene [68]. This could be a superior indicator of lipid content under nutrient deficient conditions [39, 68].

### In silico analysis

#### Gene prediction and docking

**Fig. 4:**
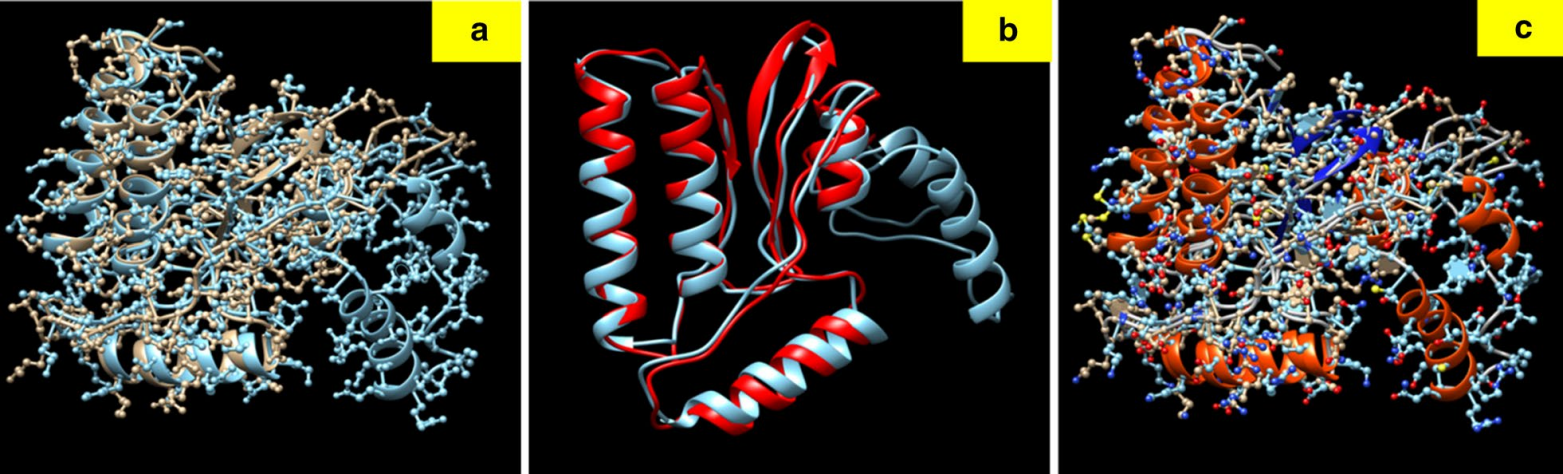
Comparison between 3D models of SP8 and SP18 proteins. **a** Ball and stick models. **b** Ribbon models. **c** Ball and stick models with different color

Predicted SP18 gene (456 bp) from *Microcoleus* sp. was smaller than SP8 gene (585 bp) from *Oscillatoria* sp. and both the genes were found to be full-length genes. Acetyl-Coenzyme A carboxyl transferase domain at N-terminal region (COA_CT_NTER) identified as an active site in both the proteins. Except first 44 amino acids, SP8 protein (45–194 aa) showed the COA_CT_NTER active site (score = 13.021), whereas in SP18 protein, the active site (score = 12.196) displayed from 1 to 151 aa. For three-dimensional (3D) structure model, both the proteins (SP8 and SP18) were submitted for ab initio as well as homology-based modeling (Additional file 1: Figures S2–S4). The obtained 3D structure of SP8 protein (21.8 kDa) showed only six alpha helices. While SP18 protein (16.7 kDa) exhibited four alpha helices and four beta sheets (Fig. 4). There was no beta sheet present in the 3D structure of the SP8 protein.

**Fig. 5:**
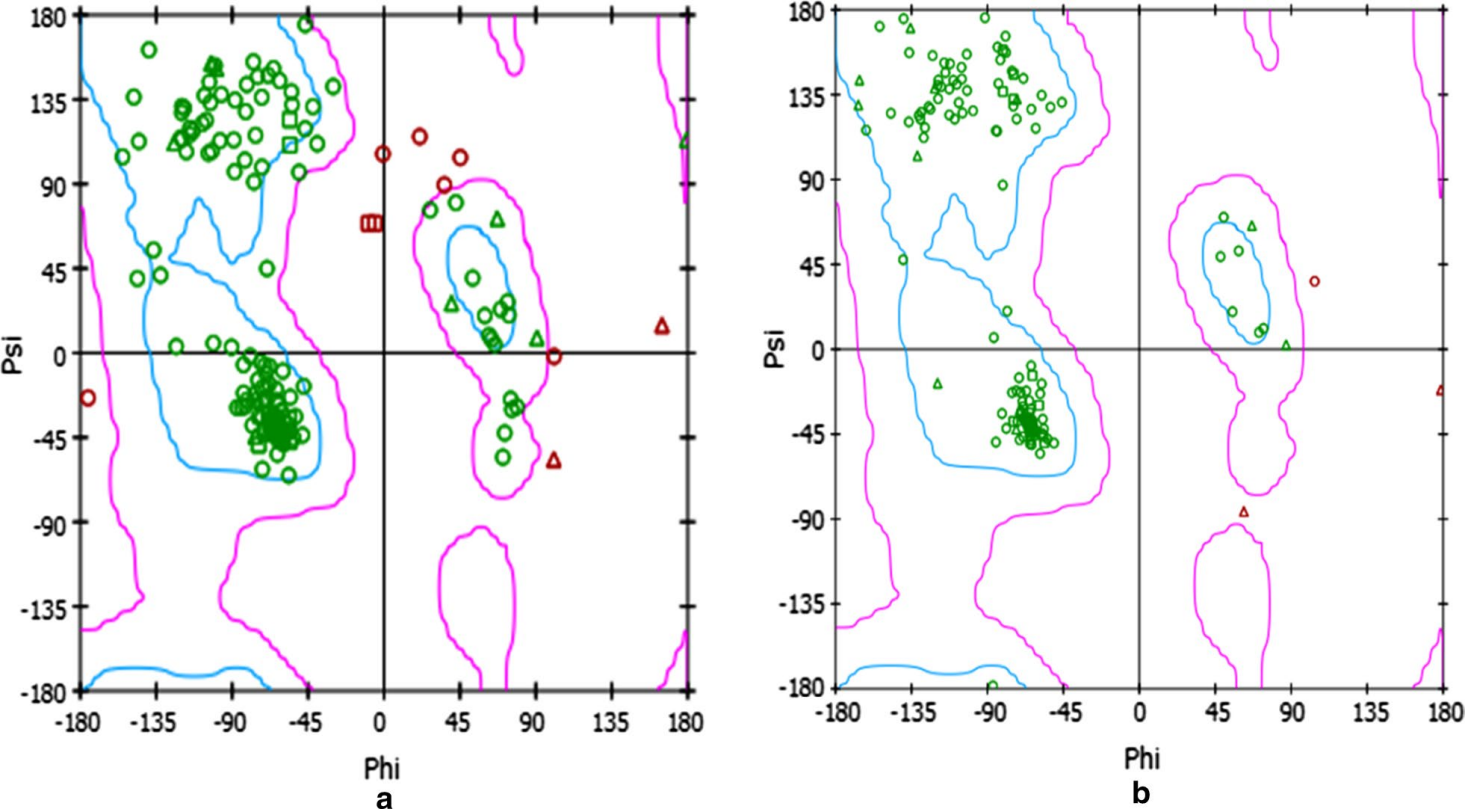
Ramachandran plot designed from the amino acid sequence of (**a**) SP8 protein and (**b**) SP18 protein

To compare both proteins i.e., SP8 and SP18 at the structural level, the SP18 protein was found more stable than SP8 protein with the occurrence of beta sheets in its secondary structure (Fig. 4). The physicochemical properties of the protein sequences of both genes, SP8 and SP18 were calculated and the amino acid compositions of the proteins are given in Additional file 1: Tables S3–S6. In this analysis, the instability index was computed for both SP8 and SP18 proteins. The instability index of SP8 and SP18 proteins was 37.21 and 30.47, respectively. They were classified as a stable protein by the software. In this analysis, similar trend was observed stating that SP18 protein is more stable than SP8 protein because it has lower instability index value than that of SP8 protein. The phi(*ϕ*)/psi(*ψ*) angles of the amino acid residues observed in Ramachandran plot analysis has shown that both proteins, SP8 and SP18, were highly stable and having more than 90% amino acids in allowed regions (Fig. 5). The allowed amino acid residues in this plot were 95.87% for SP8 protein and 98.67% for SP18 protein. The QMEAN server used to find the overall quality of three-dimensional structure of SP8 and SP18 proteins and the QMEAN score of SP8 and SP18 displayed was 0.535 and 0.464, respectively.

**Fig. 6:**
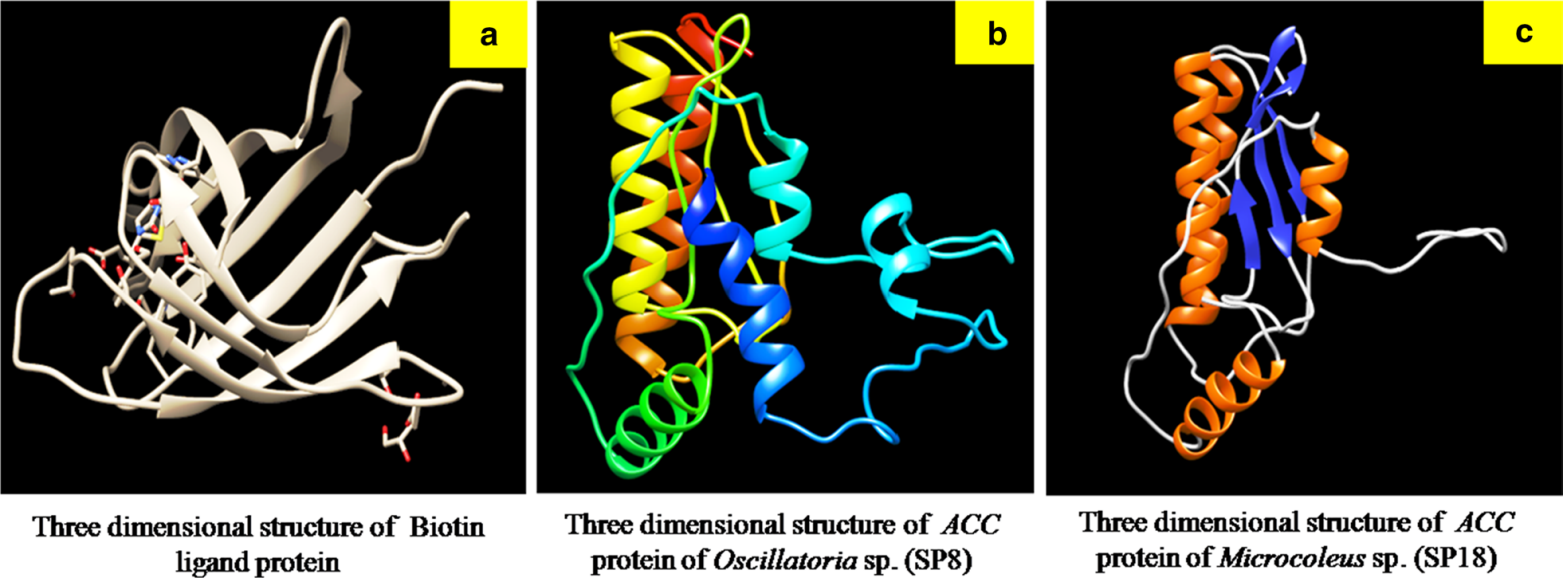
Predicted 3D models of **a** biotin ligand and *ACC* proteins of **b** *Oscillatoria* sp. (SP8) and **c** *Microcoleus* sp. (SP18)

**Fig. 7:**
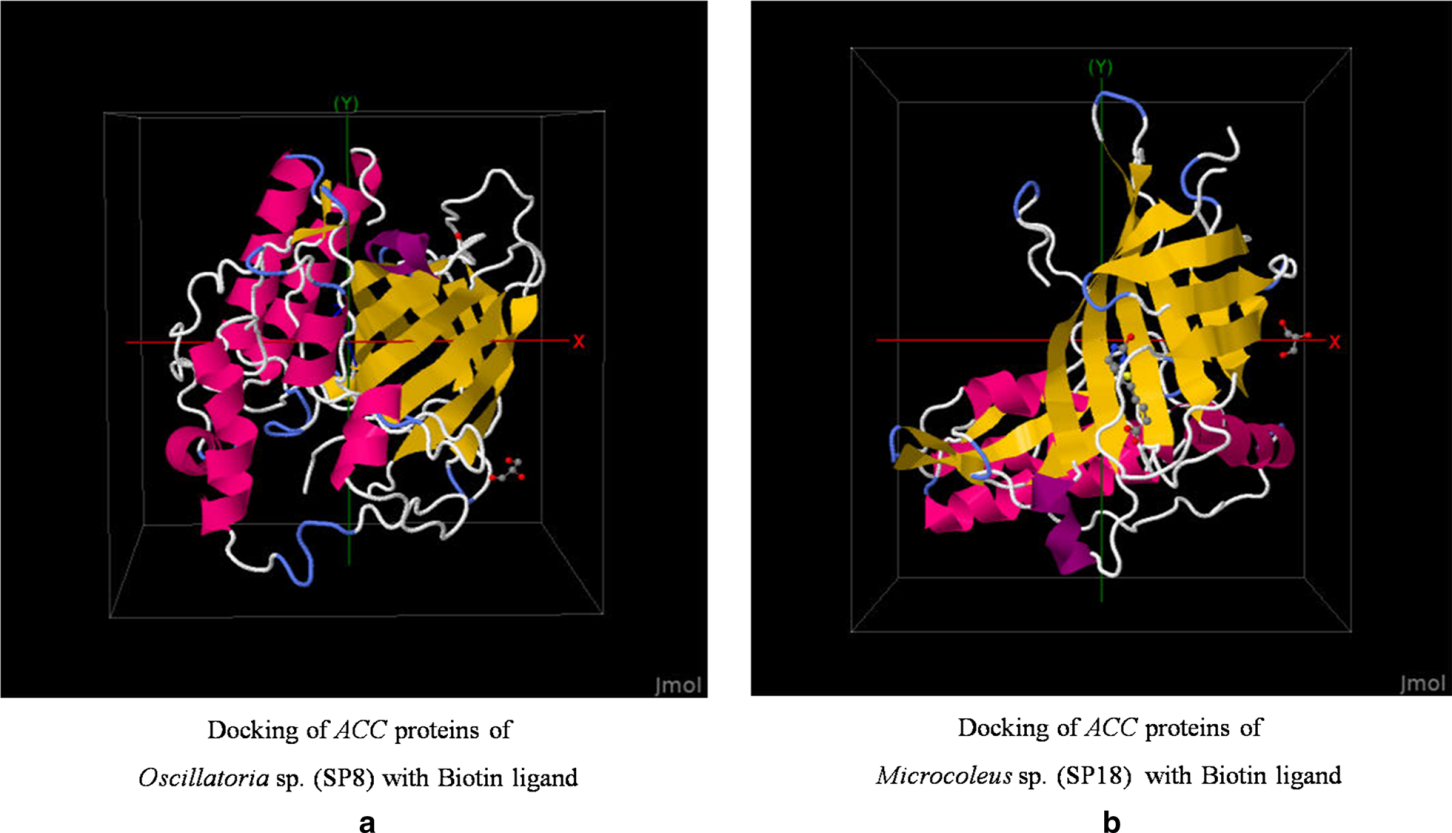
Docking of predicted 3D models. **a** Docking of *ACC* proteins of *Oscillatoria* sp. (SP8) and **b** Docking of *ACC* proteins of *Microcoleus* sp. (SP18) subjected with Biotin ligand protein

The QMEAN score gives a combined score of six different parameter viz., C_beta interaction energy, all-atom pairwise energy, solvation energy, torsion angle energy, secondary structure agreement, and solvent accessibility agreement. The estimated model reliability was in the range between 0 and 1 and they were considered as reliable 3D conformational structure. The docking results of protein–protein interaction (SP8-1C2Q; SP18-1C2Q) were obtained in the ZDOCK score. The highest ZDOCK score for a particular protein–protein binding is considered as the highest rank for their first interacting complex. This scoring function includes shape complementarity, electrostatics, and a pair wise atomic statistical potential developed using contact propensities of transient protein complexes. In our analysis, the highest ZDOCK score obtained was 1324.398 for SP8-1C2Q and 1217.939 for SP18-1C2Q. Here the protein binding affinity shown with biotin was more in case of SP8 protein than SP18 protein (Figs. 6, 7). Although the stability of SP18 protein is higher than SP8, affinity of SP18 towards Biotin is lesser than SP8. This might be due to the higher accessible vertices present in SP8 (72.81) than present in case of SP18 (63.75). The predicted local error of SP18 structure model for first 50 amino acids is higher than SP8 structure model and this might be another possible reason for their varying interacting affinity towards biotin.

### Molecular dynamics simulation

**Fig. 8:**
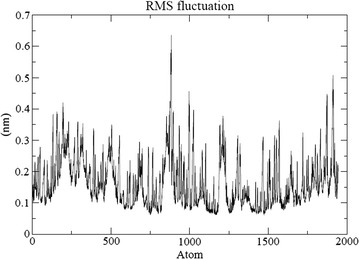
Root mean square fluctuation of atoms during the simulation process of protein ligand complex

**Fig. 9:**
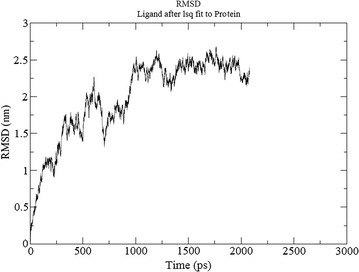
Root mean square deviation of protein ligand complex

The energy minimization of ligand-bound protein complexes was analyzed after simulations were carried out for stabilizing the same. The protein deviated about 1 Å in the first ns and then acquired a stable conformation for the rest of trajectory. RMSF per residue and Hydrogen bonds throughout simulations between protein and ligand has been calculated. A nominal change in the hydrogen bond formation between ligand and protein was observed (Fig. 8). Thus, the complex was stable for most part of the simulation trajectory. Also, root mean squared fluctuation (RMSF) is the time-average of root mean squared deviation (RMSD) for each residue (Fig. 9). In most cases, residues lying in the core protein regions have low RMSF while exposed loops have high RMSF values. As observed, the peaks in the graph possess a value between 0.2 and 0.4 nm. Both these results indicate that the ligand-bound protein complexes were stable throughout MD simulations and thus ligands possess the ability to stably bind to protein.

## Conclusion

The study revealed a time-dependent accumulation of lipids in selected cyanobacteria showing increase with increase in incubation time. The changes in the lipid content and the acetyl-Co A carboxylase (*acc*D) gene expression under nitrogen/phosphorus limitation could be correlated and provide an insight into the regulation of genes involved in lipid production pathway. The predicted 3D structure of the proteins confirmed goodness of the built model and the Instability index (Ii) of less than 40 indicated that the proteins are stable. This has been further confirmed as the ligand-bound proteins were stable throughout MD simulations. The ligand and protein were forming h-bond which indicated an effective binding. This indicated that these cyanobacteria may be stable and could be utilized for algal biofuel production on large scale.

## Additional file

**Additional file 1: Table S1.** Primers used for gene specific amplification and quantitative real time PCR analysis of Acetyl-CoA carboxylase (accD) gene (Y = C/T, R = A/G, N = A/T/G/C, H = A/C/T, W = A/T, D = A/G/T). **Table S2.** Relationship of accD gene of test organism as done by BLAST search results and the accession numbers. **Table S3.** ProtParam analysis for fetching the physicochemical parameters of predicted protein of Oscillatoria sp. (SP8). **Table S4.** Secondary structural features of SP8 protein. **Table S5.** ProtParam analysis for fetching the physicochemical parameters of predicted protein of Microcoleus sp. (SP18). **Table S6.** Secondary structural features of SP18 protein. **Figure S1.** Amplification of accD gene in SP8 and SP18. **Figure S2.** protein structure of Oscillatoria sp. (SP8). **Figure S3.** protein structure of Microcoleus sp. (SP18).

## Abbreviations

FAME: fatty acid methyl esters
ACC: acetyl-CoA carboxylase
CoA: acetyl-Coenzyme
TAG: triacylglyceride
ATP: adenosine triphosphate
NADPH: nicotinamide adenine dinucleotide phosphate
ADP: adenosine 5′-diphosphate
NADP^+^: nicotinamide adenine dinucleotide phosphate
SFA: saturated fatty acid
MUFA: mono unsaturated fatty acid
BGA: blue green algae
mM: milliMolar
DNA: deoxyribonucleic acid
RNA: ribonucleic acid
rRNA: ribosomal ribonucleic acid
cDNA: complementary DNA
PCR: polymerase chain reaction
q RT-PCR: quantitative-real-time-reverse transcription polymerase chain reaction
3D: three-dimensional
ANOVA: analysis of variance
CD: critical differences
DMRT: Duncan’s multiple range test
