# Supplementary material for: Lipid production and molecular dynamics simulation for regulation of accD gene in cyanobacteria under different N and P regimes
Source: Biotechnol Biofuels. 2017 Apr 17;10:94. doi: 10.1186/s13068-017-0776-2 (PMC5393026; doi:10.1186/s13068-017-0776-2)
Supplement: Supplementary file 1 — Additional file 1: Table S1. Primers used for gene specific amplification and quantitative real time PCR analysis of Acetyl-CoA carboxylase (accD) gene (Y = C/T, R = A/G, N = A/T/G/C, H = A/C/T, W = A/T, D = A/G/T). Table S2. Relationship of accD gene of test organism as done by BLAST search results and the accession numbers. Table S3. ProtParam analysis for fetching the physicochemical parameters of predicted protein of Oscillatoria sp. (SP8). Table S4. Secondary structural features of SP8 protein. Table S5. ProtParam analysis for fetching the physicochemical parameters of predicted protein of Microcoleus sp. (SP18). Table S6. Secondary structural features of SP18 protein. Figure S1. Amplification of accD gene in SP8 and SP18. Figure S2. protein structure of Oscillatoria sp. (SP8). Figure S3. protein structure of Microcoleus sp. (SP18). [file 13068_2017_776_MOESM1_ESM.docx]

**SUPPLEMENTARY MATERIALS**

**Supplementary Tables**

1. **Supplementary table 1** Primers used for gene specific amplification and quantitative real time PCR analysis of Acetyl-CoA carboxylase (*acc*D) gene (Y=C/T, R=A/G, N=A/T/G/C, H=A/C/T, W=A/T, D=A/G/T)
2. **Supplementary tables 2** Relationship of *acc*D gene of test organism as done by BLAST search results and the accession numbers:
3. **Supplementary table 3** ProtParam analysis for fetching the physicochemical parameters of predicted protein of *Oscillatoria* sp. (SP8).
4. **Supplementary table 4** Secondary structural features of SP8 protein.
5. **Supplementary table 5** ProtParam analysis for fetching the physicochemical parameters of predicted protein of *Microcoleus* sp. (SP18).
6. **Supplementary table 6** Secondary structural features of SP18 protein.

**Supplementary table 1 Primers used for gene specific amplification and quantitative real time PCR analysis of Acetyl-CoA carboxylase (*acc*D) gene (Y=C/T, R=A/G, N=A/T/G/C, H=A/C/T, W=A/T, D=A/G/T)**

| **S. No.** | **Primers name** | **Purpose** | **Primer sequence-Forward/Reverse**  **(5’-3’)** | **Amplicon size (bp)** | **Melting Point (^o^C)** |
| --- | --- | --- | --- | --- | --- |
| 1. | SP8-PCR | Standard PCR | F: ATGTCYYTRTTYGATTGGTT  R: CGNTCRAAACGRTACRAHCC | 950 | 58 |
| 2. | SP18-PCR | Standard PCR | F: ATGGCWAACAACGAAGARTC  R:GDATYTTNGCCATYTGCAT | 1150 | 56 |
| 3. | SP8-qPCR | Real Time PCR | F:AGTCCCGAACGAATTCAACAA  R:CACCAGAAGCACAGACAATA | 107 | 62 |
| 4. | SP18-qPCR | Real Time PCR | F:AGTCCCGAACGAATTCAACAA  R:CGCTATATTGCTTGCGGTCT | 109 | 62 |

| **Supplementary table 2 Relationship of *acc*D gene of test organism as done by BLAST search results and the accession numbers:** | | |  |
| --- | --- | --- | --- |
| **Cyanobacterial Species** | **Gene** | **Accession No.** |  |
| *Oscillatoria* sp.  (SP8) | beta subunit gene for acetyl- Co A carboxylase, Carboxyl transferase | LN606590 |  |
| *Microcoleus* sp. (SP18) | beta subunit gene for acetyl- Co A carboxylase, Carboxyl transferase | LN606589 |  |
|  | | | |

**Supplementary table 3 ProtParam analysis for fetching the physicochemical parameters of predicted protein of *Oscillatoria* sp. (SP8).**

| \| ***Amino acid composition, frequency & percentage*** \| \| \| \| \| \| --- \| --- \| --- \| --- \| --- \| \| **S.No** \| **!** \| ***** \| **#** \| **$** \| \| 1 \| Ala \| (A) \| 9 \| 4.60% \| \| 2 \| Arg \| (R) \| 13 \| 6.70% \| \| 3 \| Asn \| (N) \| 4 \| 2.10% \| \| 4 \| Asp \| (D) \| 12 \| 6.20% \| \| 5 \| Cys \| (C) \| 7 \| 3.60% \| \| 6 \| Gln \| (Q) \| 17 \| 8.80% \| \| 7 \| Glu \| (E) \| 12 \| 6.20% \| \| 8 \| Gly \| (G) \| 15 \| 7.70% \| \| 9 \| His \| (H) \| 2 \| 1.00% \| \| 10 \| Ile \| (I) \| 8 \| 4.10% \| \| 11 \| Leu \| (L) \| 18 \| 9.30% \| \| 12 \| Lys \| (K) \| 12 \| 6.20% \| \| 13 \| Met \| (M) \| 9 \| 4.60% \| \| 14 \| Phe \| (F) \| 4 \| 2.10% \| \| 15 \| Pro \| (P) \| 9 \| 4.60% \| \| 16 \| Ser \| (S) \| 13 \| 6.70% \| \| 17 \| Thr \| (T) \| 10 \| 5.20% \| \| 18 \| Trp \| (W) \| 3 \| 1.50% \| \| 19 \| Tyr \| (Y) \| 4 \| 2.10% \| \| 20 \| Val \| (V) \| 13 \| 6.70% \| |  |
| --- | --- | --- | --- | --- | --- | --- | --- | --- | --- | --- | --- | --- | --- | --- | --- | --- | --- | --- | --- | --- | --- | --- | --- | --- | --- | --- | --- | --- | --- | --- | --- | --- | --- | --- | --- | --- | --- | --- | --- | --- | --- | --- | --- | --- | --- | --- | --- | --- | --- | --- | --- | --- | --- | --- | --- | --- | --- | --- | --- | --- | --- | --- | --- | --- | --- | --- | --- | --- | --- | --- | --- | --- | --- | --- | --- | --- | --- | --- | --- | --- | --- | --- | --- | --- | --- | --- | --- | --- | --- | --- | --- | --- | --- | --- | --- | --- | --- | --- | --- | --- | --- | --- | --- | --- | --- | --- | --- | --- | --- | --- | --- |
|  | |
|  | |

**!** Amino acid three letter

***** Amino acid single letter

**#** Frequency of particular amino acid

**$** Percentage of particular amino acid

| **Supplementary table 4 Secondary structural features of SP8 protein.** |
| --- |
| \| ***Number of alpha and beta helices sheets*** \| \| Start position \| End position \| \| --- \| --- \| --- \| --- \| \| alpha helices sheet \| 1st \| LYS 4 \| GLN 16 \| \|  \| 2nd \| THR 37 \| ASN 43 \| \|  \| 3rd \| SER 58 \| GLN 64 \| \|  \| 4th \| ASP 88 \| ASP 101 \| \|  \| 5th \| SER 137 \| GLN 152 \| \|  \| 6th \| GLY 171 \| SER 190 \| |

**Supplementary table 5 ProtParam analysis for fetching the physicochemical parameters of predicted protein of *Microcoleus* sp.(SP18).**

| \| ***Amino acid composition, frequency & percentage*** \| \| \| \| \| \| --- \| --- \| --- \| --- \| --- \| \| **S.No** \| **!** \| ***** \| **#** \| **$** \| \| 1 \| Ala \| (A) \| 8 \| 5.30% \| \| 2 \| Arg \| (R) \| 11 \| 7.30% \| \| 3 \| Asn \| (N) \| 2 \| 1.30% \| \| 4 \| Asp \| (D) \| 10 \| 6.60% \| \| 5 \| Cys \| (C) \| 4 \| 2.60% \| \| 6 \| Gln \| (Q) \| 11 \| 7.30% \| \| 7 \| Glu \| (E) \| 8 \| 5.30% \| \| 8 \| Gly \| (G) \| 15 \| 9.90% \| \| 9 \| His \| (H) \| 2 \| 1.30% \| \| 10 \| Ile \| (I) \| 7 \| 4.60% \| \| 11 \| Leu \| (L) \| 13 \| 8.60% \| \| 12 \| Lys \| (K) \| 9 \| 6.00% \| \| 13 \| Met \| (M) \| 9 \| 6.00% \| \| 14 \| Phe \| (F) \| 4 \| 2.60% \| \| 15 \| Pro \| (P) \| 10 \| 6.60% \| \| 16 \| Ser \| (S) \| 8 \| 5.30% \| \| 17 \| Thr \| (T) \| 6 \| 4.00% \| \| 18 \| Trp \| (W) \| 1 \| 0.70% \| \| 19 \| Tyr \| (Y) \| 2 \| 1.30% \| \| 20 \| Val \| (V) \| 11 \| 7.30% \| |  |
| --- | --- | --- | --- | --- | --- | --- | --- | --- | --- | --- | --- | --- | --- | --- | --- | --- | --- | --- | --- | --- | --- | --- | --- | --- | --- | --- | --- | --- | --- | --- | --- | --- | --- | --- | --- | --- | --- | --- | --- | --- | --- | --- | --- | --- | --- | --- | --- | --- | --- | --- | --- | --- | --- | --- | --- | --- | --- | --- | --- | --- | --- | --- | --- | --- | --- | --- | --- | --- | --- | --- | --- | --- | --- | --- | --- | --- | --- | --- | --- | --- | --- | --- | --- | --- | --- | --- | --- | --- | --- | --- | --- | --- | --- | --- | --- | --- | --- | --- | --- | --- | --- | --- | --- | --- | --- | --- | --- | --- | --- | --- | --- |

**!** Amino acid three letter

***** Amino acid single letter

**#** Frequency of particular amino acid

**$** Percentage of particular amino acid

| **Supplementary table 6 Secondary structural features of SP18 protein**. |
| --- |
| \| ***Number of alpha and beta helices sheets*** \| \| start position \| end position \| \| --- \| --- \| --- \| --- \| \| alpha helices sheet \| 1st \| SER 14 \| LEU 21 \| \|  \| 2nd \| ARG 45 \| LYS 58 \| \|  \| 3rd \| SER 93 \| LYS 109 \| \|  \| 4th \| MET 128 \| ARG 146 \| \| beta helices sheet \| 1st \| VAL 65 \| GLY 68 \| \|  \| 2nd \| GLY 70 \| LEU 72 \| \|  \| 3rd \| LEU 75 \| MET 82 \| \|  \| 4th \| VAL 113 \| VAL 116 \| |

**Supplementary Figures**

1. **Supplementary Fig. 1** Amplification of *acc*D gene in SP8 and SP18.
2. **Supplementary Fig. 2** 3D protein structure of *Oscillatoria* sp. (SP8).
3. **Supplementary Fig. 3** 3D protein structure of *Microcoleus* sp.(SP18).

**Supplementary Fig.1** Amplification of *acc*D gene.


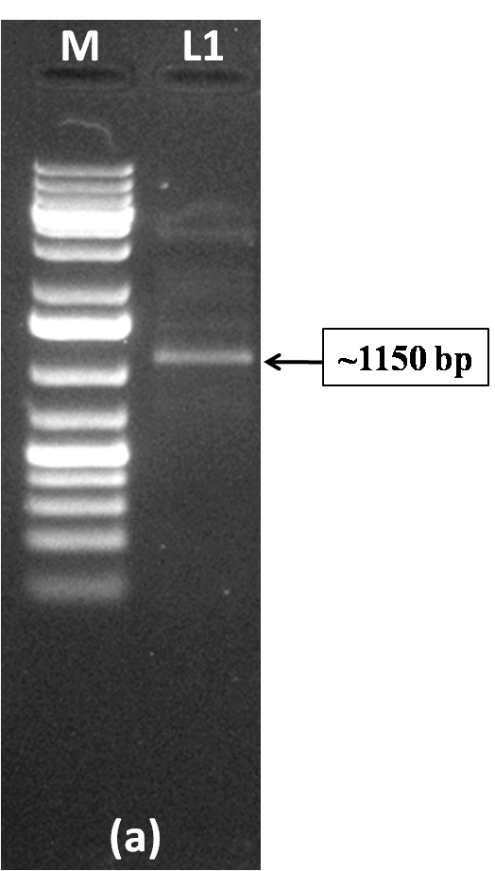

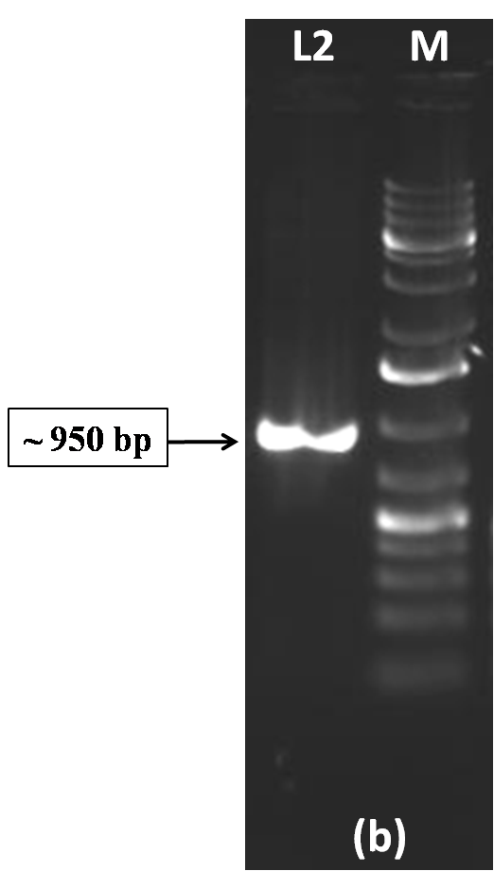


**Supplementary Fig. 2** 3D protein structure of *Oscillatoria* sp. (SP8).

**
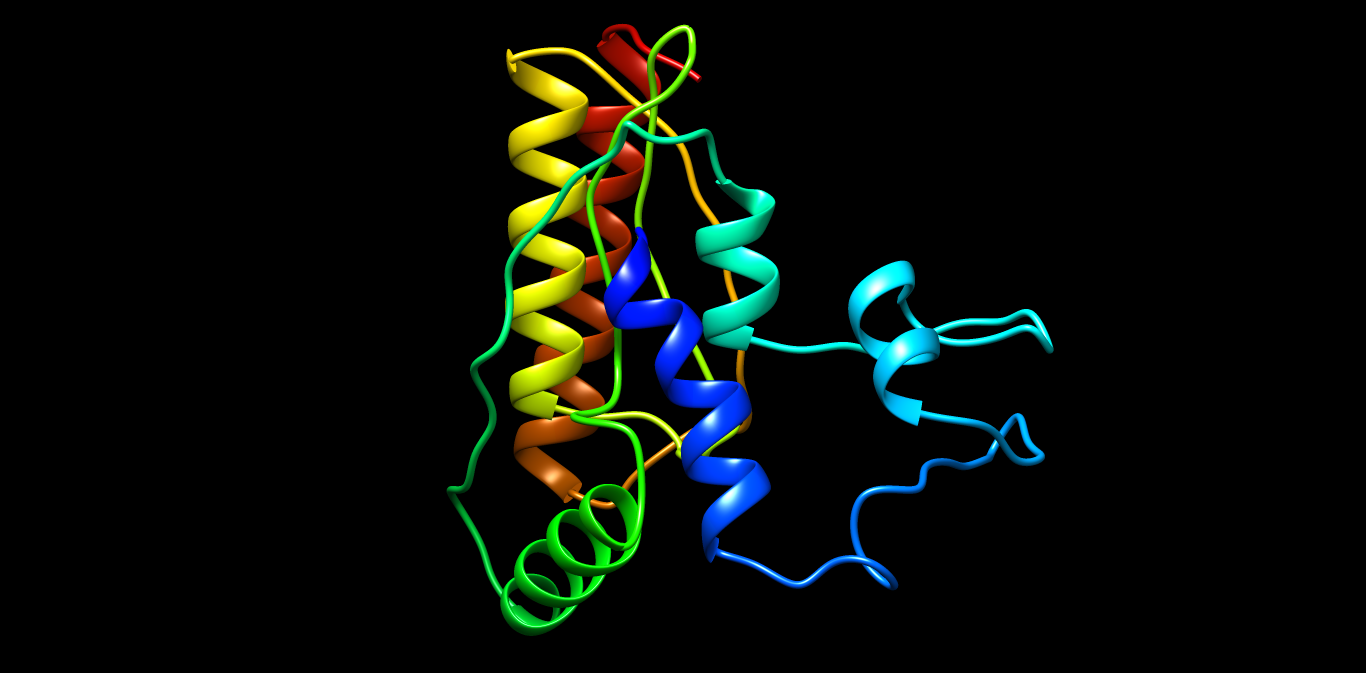
**

**Supplementary Fig. 3** 3D protein structure of *Microcoleus* sp. (SP18).

**
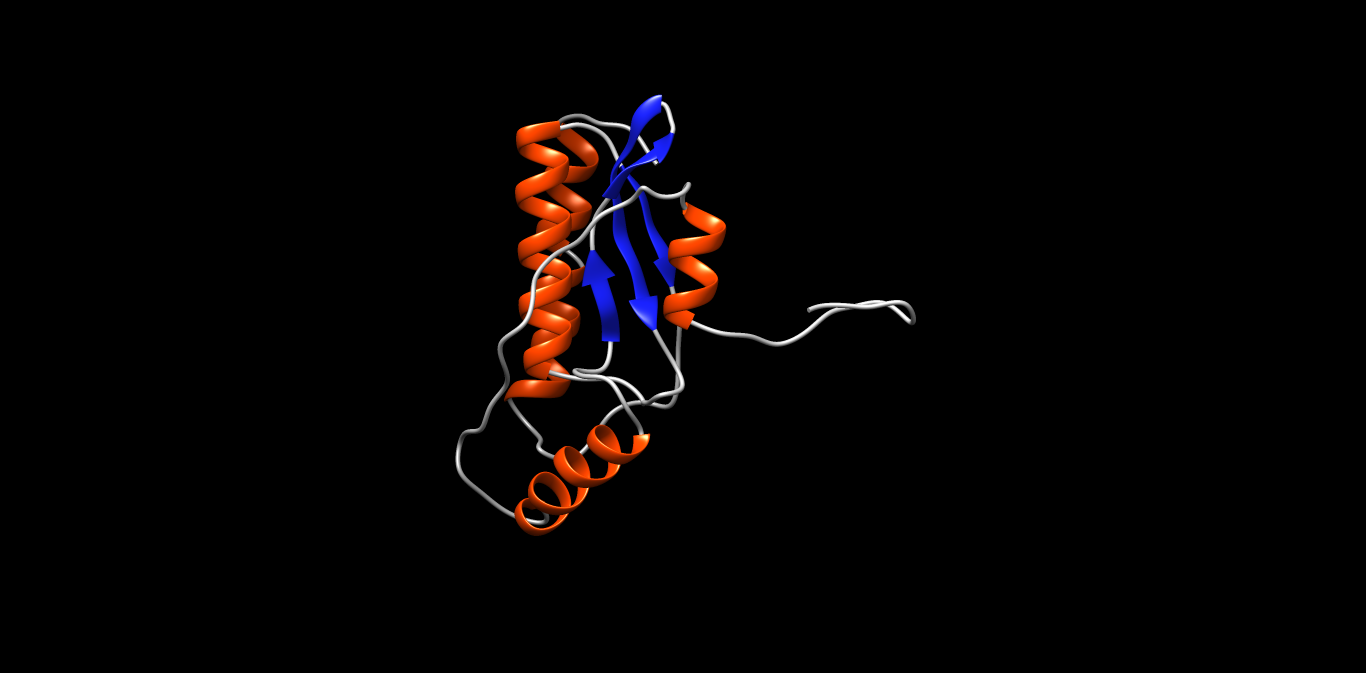
**
